# Is neuron-specific enolase useful for diagnosing malignant pleural effusions? evidence from a validation study and meta-analysis

**DOI:** 10.1186/s12885-017-3572-2

**Published:** 2017-08-30

**Authors:** Jing Zhu, Mei Feng, Liqun Liang, Ni Zeng, Chun Wan, Ting Yang, Yongchun Shen, Fuqiang Wen

**Affiliations:** Department of Respiratory and Critical Care Medicine, West China Hospital of Sichuan University and Division of Pulmonary Diseases, State Key Laboratory of Biotherapy of China, Chengdu, 610041 China

**Keywords:** Neuron-specific enolase, Malignant pleural effusion, Diagnosis, Meta-analysis

## Abstract

**Background:**

Neuron-Specific enolase (NSE) has been used as a typical tumor marker and shows a potential to diagnose malignant pleural effusion (MPE). The ability of NSE in diagnosing MPE has been investigated in many studies, but with inconsistent conclusions. This study sought to investigate the diagnostic accuracy of NSE for MPE through a clinical study and together with a meta-analysis.

**Methods:**

Pleural effusion samples from 136 patients with MPE and 102 patients with benign pleural effusion (BPE) were collected, and NSE levels were measured by electrochemiluminescence immunoassay. Receiver operating characteristic (ROC) curve analysis was performed to assess the ability of NSE to differentiate MPE from BPE. Literature search was conducted to identify suitable publications, data were extracted and diagnostic indexes including sensitivity, specificity, positive/negative likelihood ratio (PLR/NLR), and diagnostic odds ratio (DOR) were pooled. Summary ROC curve was generated to determine the overall diagnostic accuracy of NSE for MPE.

**Results:**

Levels of NSE were significantly increased in pleural effusion from patients with MPE than that from BPE (18.53 ± 27.30 vs. 6.41 ± 6.95 ng/ml, *p* < 0.001). With a cut-off value of 8.92 ng/ml, pleural NSE had a sensitivity of 59.56% and a specificity of 83.33% in diagnosing MPE. A total of 14 studies with 1896 subjects were included for meta-analysis. The diagnostic parameters of NSE were listed as follows: sensitivity, 0.53 (95% CI: 0.38–0.67); specificity, 0.85 (95% CI: 0.75–0.91); PLR, 3.54 (95% CI: 2.33–5.39); NLR, 0.56 (95% CI: 0.42–0.73); and DOR, 6.39 (95% CI: 3.72–10.96). The area under the summary ROC curve was 0.78.

**Conclusions:**

The role of pleural NSE measurement in diagnosing MPE is limited and with a low sensitivity. The clinical utility of NSE assay should be combined with the results of other tumor markers examination and the detail clinical information of patient. Further studies are needed to confirm the role of NSE in diagnosing MPE.

**Electronic supplementary material:**

The online version of this article (10.1186/s12885-017-3572-2) contains supplementary material, which is available to authorized users.

## Background

Neuron-specific enolase (NSE), which localized predominately in the cytoplasm of neurons, is a cell specific isoenzyme of the glycolytic enzyme enolase [1]. During normal condition, NSE is not secreted. While NSE is up-regulated to maintain homeostasis when axons are injured, thus, NSE is a classical biomarker that directly evaluates functional damage to neurons [2], and lots of studies have found that NSE is a biomarker of neurological disorders [3]. Considering NSE as a specific biomarker for neurons and peripheral neuroendocrine tissues, the increased expression of NSE in both tissues and circulations may be presented with malignant proliferation of neuroendocrine tissues, and thus could be of potential value in the diagnosing, staging and guiding treatment of such cancers [1, 4].

Small-cell lung cancer (SCLC), a malignant disease associated with neuroendocrine differentiation, is characterized by its rapid doubling time, high growth fraction, and early propensity for metastases [5, 6]. Non-small-cell lung cancer (NSCLC) also presented with neuroendocrine properties, since both SCLC and NSCLC originate from a common cell lineage and differentiated lately for oncogenetic development, studies reported that about 11.7–28% of patients with NSCLC presented with increased serum NSE levels [7, 8]. Thus, neuroendocrine marker like NSE has been proved to be useful in immunohistochemically differentiating NSCLC and SCLC, which released into the blood and body fluid, can be used as tumor marker [1].

Malignant pleural effusion (MPE) is caused by lung cancer and other malignant diseases. The presence of pleural effusion also suggests metastases of tumor, indicating an unoptimistic prognosis [9]. Thus, to diagnose MPE early and accurately may benefit patient with timely and effective treatments [10]. Many studies have reported that NSE levels increased significantly in MPE, NSE may be a biomarker for MPE [11, 12]. However, the results of these studies are so different, and there is no definite conclusion on the diagnostic value of NSE for MPE. The present study sought to validate the diagnostic accuracy of NSE for MPE in Chinese patients, and summarize the overall diagnostic accuracy of NSE for MPE through a meta-analysis based on current available literatures.

## Method

### Patient inclusion

Ethics Committee of West China Hospital of Sichuan University approved this study protocol. This study was performed based on the principles expressed in the Declaration of Helsinki. Written informed consents were collected from all patients for the collection of clinical samples and subsequent analysis at admission.

From February 2011 to August 2013, 238 patients with undiagnosed pleural effusion admitted to our hospital for further investigation were included this retrospective clinical study. Among them, 136 patients were diagnosed as MPE, which was diagnosed by experienced pathologists based on identification of malignant cells in pleural fluid as detected using cytological tests or biopsy analysis on pleura or lung tissues. 102 patients with benign pleural effusion (BPE) were also recruited as controls.

### Sample collection and measurement

All include patients underwent a standard thoracocentesis before the treatment, during which pleural effusion samples were collected. When multiple thoracenteses were performed on the same patient, only the first sample was analyzed. For serum sample collection, after fast overnight from 21:00, venous blood samples from patients were collected and serum was separated immediately. Both pleural effusion and serum samples were collected and sent for biochemical analysis in the department of laboratory medicine. Serum and pleural NSE levels were measured by an electrochemiluminescence immunoassay (Roche Cobas 8000 modular analyser series; Roche Diagnostics, USA). Pleural glucose, total protein, lactate dehydrogenase levels were examined simultaneously. Technicians processing pleural effusion samples for NSE measurement and biochemical assays were blinded to patient details.

### Statistical analysis

Data were presented as the Means ±standard deviation. Difference in MPE and BPE groups was analyzed by the nonparametric Mann-Whitney U-test. Differences among multiple groups were detected with analysis of variance (ANOVA). Receiver operating characteristic (ROC) curves were constructed, and areas under the curve (AUC) were measured to quantify the accuracy of NSE to discriminate MPE from BPE. The optimal cut-off value was set to obtain the best sensitivity and specificity for diagnosing MPE. Statistical analysis was performed using SPSS 18.0 software (Chicago, IL, USA). A value of *p* < 0.05 was set as significant.

### Meta-analysis

This meta-analysis was carried out based on the standard method that recommend for meta-analysis of diagnostic studies and the guidelines of the Preferred Reporting Items for Systematic Reviews [13].

We searched in PubMed and EMBASE for eligible articles published up to March 2016, the following search terms were used as Medical Headings and/or text words: “Neuron specific enolase or NSE” AND “Malignant pleural effusion or malignant pleural fluid or malignant hydrothorax” AND “sensitivity or specificity or accuracy”. Potential related studies were also checked from the reference lists of the included original and review articles. Studies were included if: they measured the accuracy of pleural NSE for differentiating MPE and BPE in humans; they presented sufficient data to calculate true positive (TP), false positive (FP), false negative (FN), and true negative (TN) rates, and they were published in English. Data were retrieved and formed a 2 × 2 table of diagnostic performance. A 14-items Quality Assessment of Diagnostic Accuracy Studies (QUADAS) list was used to evaluate the quality of included studies [14].

The meta-analysis was carried out using a bivariate regression model [15, 16], with which we calculated pooled sensitivity, specificity, positive/negative likelihood ratios (PLR/NLR), and diagnostic odds ratios (DOR). We also generated summary receiver operating characteristic (SROC) curves to summarize the diagnostic accuracy performance of NSE [17]. Heterogeneity was evaluated using the I^2^ inconsistency test, I^2^ > 50% suggested substantial heterogeneity. Potential publication bias was detected by Deeks’s funnel plot test [18]. All statistical analysis was conducted using STATA 12.0 (Stata Corp., College Station, TX). All statistical analysis was two-sided, a *p* value <0.05 was set as statistically significant.

## Results

### General clinical data of patients

There were 136 patients with MPE, including 74 males and 62 females with mean ages of 58 years. In MPE patients, Cytology examinations were positive in 56 cases, corresponding to a positive rate of 41.17%. Among patients with MPE, 101 had NSCLC (90, lung adenocarcinoma; 11, lung squamous cell carcinoma); 11, small cell lung carcinoma; 18, metastatic carcinoma; 5, lymphoma, and 1, malignant mesothelioma.

There were 102 patients with BPE as controls, including 68 males and 34 females, with mean ages of 56 years. These patients had been diagnosed with the following conditions: tuberculous pleurisy, 49; parapneumonic effusion, 26; heart failure, 25; liver cirrhosis, 1; and chylothorax, 1. The MPE and BPE groups didn’t differ significantly on age or gender. The clinical information and pleural fluid characteristics of both MPE and BPE group are listed in Table 1.

**Table 1:** The demographics characteristics and biochemical results of patients

	Benign pleural effusion	Malignant pleural effusion	*p* value
No. of Patient	102	136	
Sex(male/female)	68/34	74/62	0.056
Age (years)	56 ± 19	58 ± 13	0.167
Pleural NSE (ng/ml)	6.41 ± 6.95	18.53 ± 27.30	<0.001
Pleural NSE (ng/mg of pleural protein)	0.15 ± 0.16	0.51 ± 0.88	<0.001
Pleural protein(g/l)	42.02 ± 13.04	41.84 ± 10.62	0.906
Pleural glucose (mmol/l)	5.84 ± 1.71	5.48 ± 2.51	0.216
Pleural LDH (U/l)	256.57 ± 181.22	502.99 ± 414.15	<0.001
Pleural LDH (U/g of pleural protein)	5.79 ± 3.95	13.68 ± 14.96	<0.001
Serum NSE (ng/ml)	13.77 ± 13.33	19.51 ± 16.54	0.004
Serum NSE (ng/mg of pleural protein)	0.39 ± 0.63	0.54 ± 0.61	0.067
Pleural/serum NSE ratio	0.56 ± 0.55	1.08 ± 1.54	0.001

*LDH* Lactic Dehydrogenase, *NSE* Neuron-specific enolase

### Levels of NSE

**Fig. 1:**
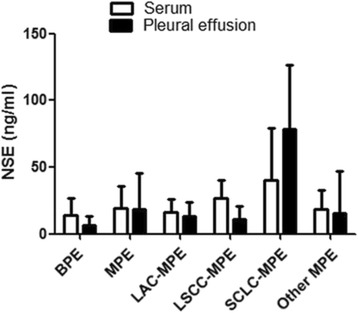
Serum and pleural levels of neuron specific enolase in patients. NSE: Neuron specific enolase; BPE: Benign pleural effusion; MPE: Malignant pleural effusion; LAC-MPE: Lung adenocarcinoma-malignant pleural effusion; LSCC-MPE: Lung squamous cell carcinoma- malignant pleural effusion; SCLC-MPE: Small cell lung cancer- malignant pleural effusion

The levels of NSE in both serum and pleural effusion were significantly increased in patients with MPE than in patients with BPE (serum 19.51 ± 16.54 vs. 13.77 ± 13.33 ng/ml, *p* = 0.004; pleural effusion 18.53 ± 27.30 vs. 6.41 ± 6.95 ng/ml, *p* < 0.001) (Table 1). In patients with MPE, the SCLC patients showed the highest levels of NSE in both serum and pleural effusion when compared with other causes of MPE (both *P* < 0.001), as shown in Fig. 1. After adjusted by pleural protein, the patients with MPE remained have a higher levels of NSE in serum and pleural effusion than patients with BPE (Additional file 1: Fig. S1).

### Diagnostic accuracy of NSE

**Fig. 2:**
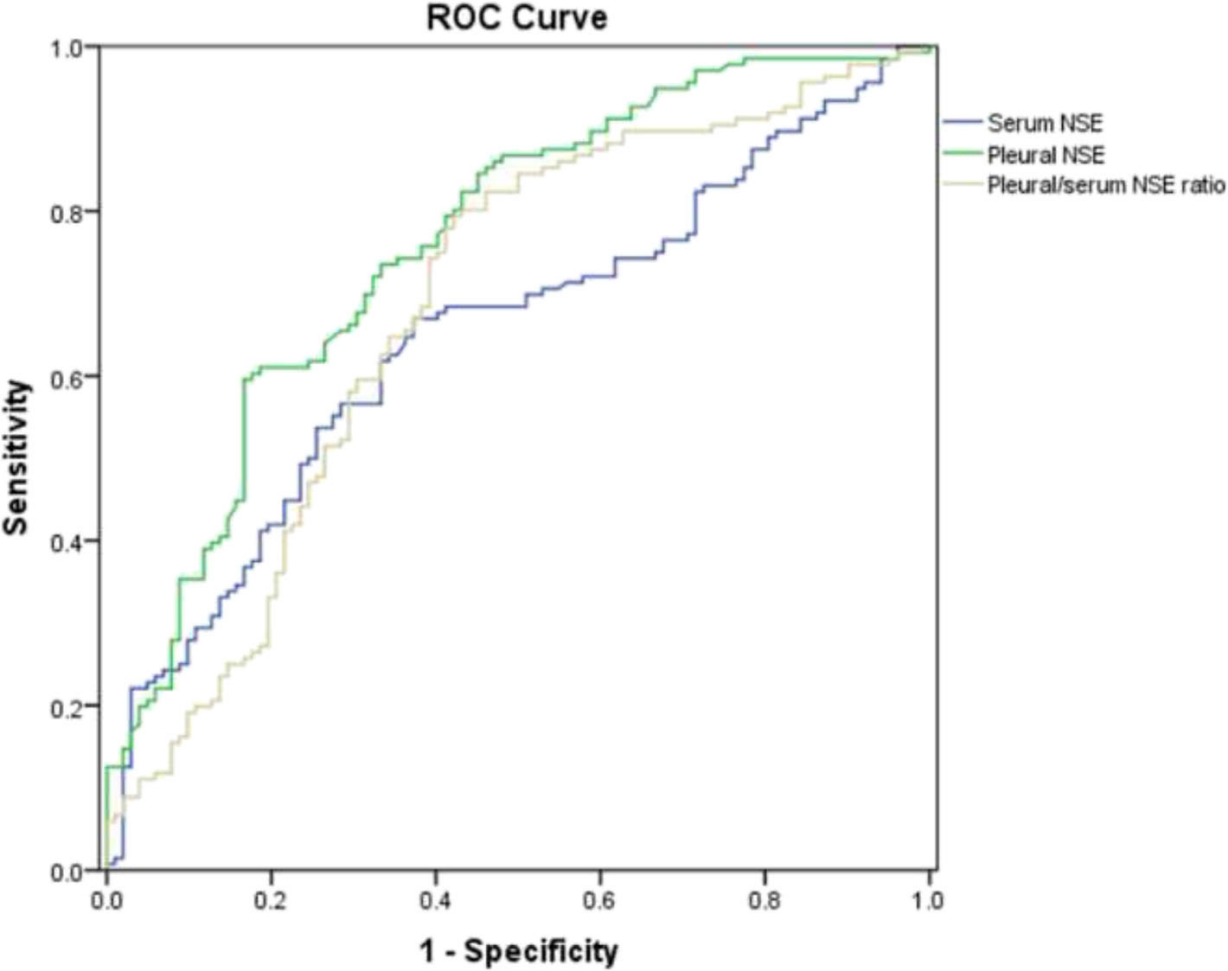
Receiver operating characteristic curve for neuron specific enolase in serum and pleural effusion for distinguishing between malignant and benign pleural effusions. NSE: Neuron specific enolase; ROC: Receiver operating characteristic

Next, we evaluated the diagnostic accuracy of NSE for MPE with ROC curves. At a cut off value of 8.92 ng/ml, the diagnostic sensitivity and specificity of pleural NSE for MPE were 59.56% and 83.33%, respectively, and the AUC was 0.76. At a cut off value of 12.29 ng/ml, serum levels of NSE play a role in diagnosing MPE with the sensitivity and specificity of 66.91% and 62.75%, respectively, but the AUC was only 0.65, as shown in Fig. 2. Meanwhile, the AUC of pleural/serum NSE ratio in diagnosing MPE was 0.68 (Fig. 2).

We also noticed that in 11 SCLC patients, the serum/pleural levels of NSE were the highest among all causes of MPE. When compared with BPE patients, at a cut-off value of 17.42 ng/ml, pleural NSE plays a valuable role in diagnosing MPE with the sensitivity and specificity of 100% and 92.16%, respectively, and the AUC was 0.99. The diagnostic summary of serum and pleural levels of NSE for MPE and SCLC related MPE was listed in Table 2.

**Table 2:** Diagnostic summary of NSE for malignant pleural effusion

	MPE			SCLC-MPE		
	Serum NSE	Pleural NSE	Pleural/serum NSE ratio	Serum NSE	Pleural NSE	Pleural/serum NSE ratio
Cut-off	12.29 ng/ml	8.92 ng/ml	0.39	12.29 ng/ml	17.42 ng/ml	0.60
Sensitivity	66.91%	59.56%	79.41%	100%	100%	100%
Specificity	62.75%	83.33%	57.84%	62.75%	92.16%	69.60%
AUC	0.65	0.76	0.68	0.86	0.99	0.92

*AUC* Area under the curve, *MPE* Malignant pleural effusion, *NSE* Neuron-specific enolase, *SCLC* Small cell lung cancer

### Meta-analysis

This meta-analysis included 14 studies (including present study), consisting 1093 cases of MPE and 803 BPE controls [19–31]. All the MPEs were diagnosed based on cytology and histology examinations, which were widely accepted as the gold standard for MPE diagnosis. There were 11 studies with QUADAS score ≥ 9, indicating the reliability of statistical results. The clinical summary of individual study and QUADAS score were listed in Table 3.

**Table 3:** Clinical summary of included studies

First author	Year	Country	Cases/controls	Standard	Method	Cut-off value	TP	FP	FN	TN	QUADAS
Pettersson T	1988	Finland	31/22	Cytology, Histology	Radioimmunoassay	12.5μg/L	10	4	21	18	7
Shimokata K	1989	Japan	59/39	Cytology, Histology	EIA	26 ng/ml	11	2	48	37	8
Menard O	1993	France	24/18	Histology	Radioimmunoassay	8 ng/ml	13	2	11	16	8
San Jose ME	1997	Spain	88/183	Cytology, Histology	EIA	8.8μg/L	26	21	62	162	9
Miédougé M	1999	France	215/121	Cytology, Histology	EIA	18.1 ng/ml	39	3	176	118	10
Kuralay F	2000	Turkey	21/40	Histology	ELISA	8.7 ng/ml	21	2	0	38	9
Lee JH	2005	Korea	34/16	Histology	ELISA	20 ng/ml	12	1	22	15	10
Ghayumi SM	2005	Iran	40/37	Cytology, Histology	ELISA	5.21μg/ml	27	9	13	28	10
Topolcan O	2007	Czech Republic	80/78	Cytology, Histology	Immuno-radiometric assay	13.1 ng/ml	34	4	46	74	9
Wu GP	2007	China	74/34	Cytology, Histology	Immunoassay	5.2μg/L	51	15	23	19	9
Korczynski P	2009	Poland	36/38	Cytology, Histology	ECLIA	0.22 ng/ml	34	24	2	14	10
Wang Y	2013	China	160/40	Cytology, Histology	ECLIA	NA	95	15	65	25	11
Gu Y	2016	China	95/35	Histology	ECLIA	9.71 ng/ml	50	8	45	27	11
Zhu J	2016	China	136/102	Cytology, Histology	ECLIA	8.92 ng/ml	81	17	55	85	10

*EIA* Enzyme immunoassay, *ECLIA* Electrochemiluminescence immunoassay, *ELISA* Enzyme linked immunosorbent assay, *FN* false negative, *FP* false positive, *NA* Not available, *QUADAS* Quality Assessment of Diagnostic Accuracy Studies, *TN* true negative, *TP* true positive

**Fig. 3:**
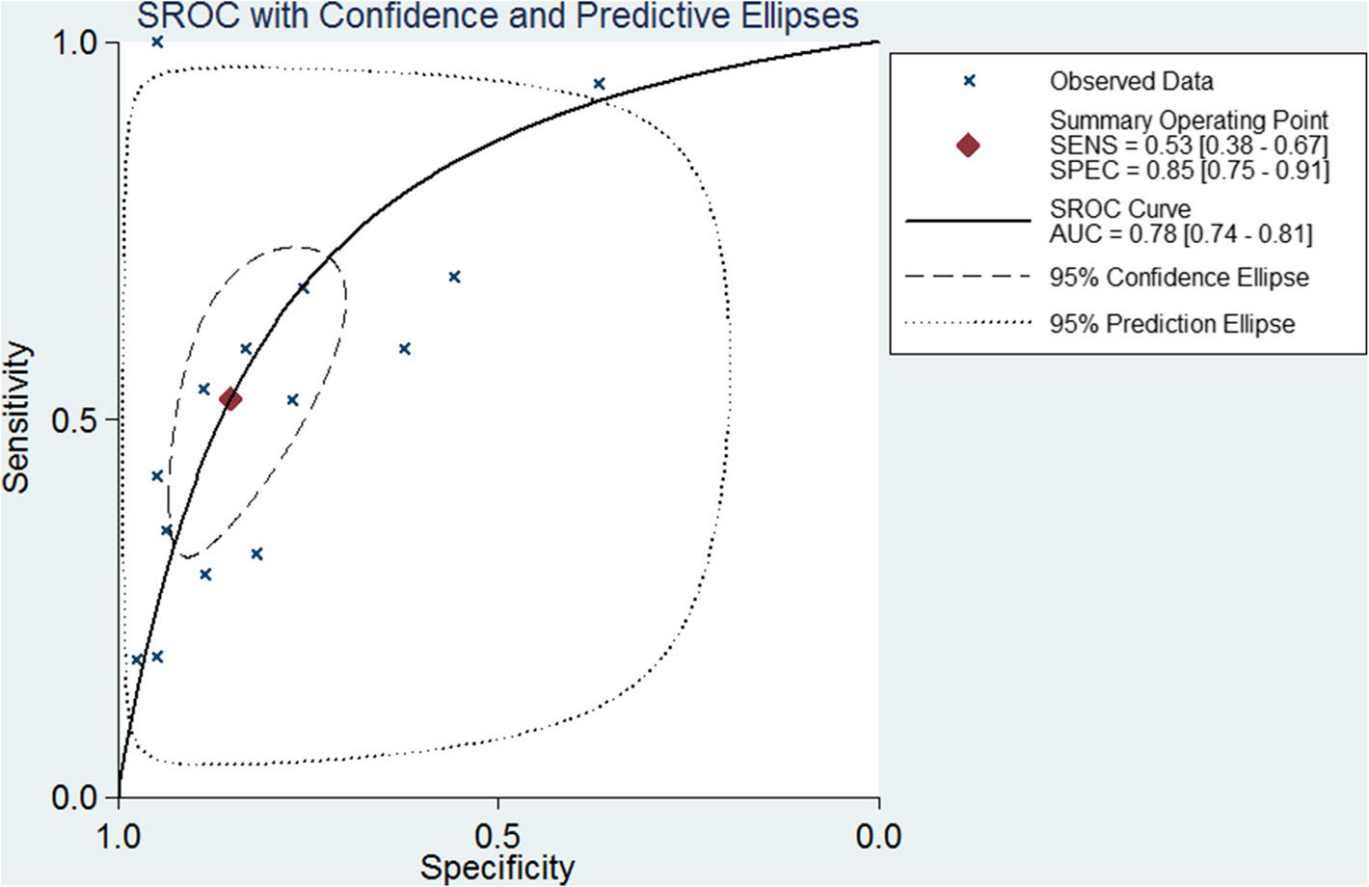
Summary receiver operating characteristic (SROC) curve for pleural neuron specific enolase tests. AUC: Area under the curve

The pooled parameters for pleural NSE in diagnosing MPE over all 14 studies were listed as follows: sensitivity, 0.53 (95% CI: 0.38–0.67); specificity, 0.85 (95% CI: 0.75–0.91); PLR, 3.54 (95% CI: 2.33–5.39); NLR, 0.56 (95% CI: 0.42–0.73); and DOR, 6.39 (95% CI: 3.72–10.96). Figure 3 showed the corresponding SROC curve, which yield an AUC of 0.78.

**Fig. 4:**
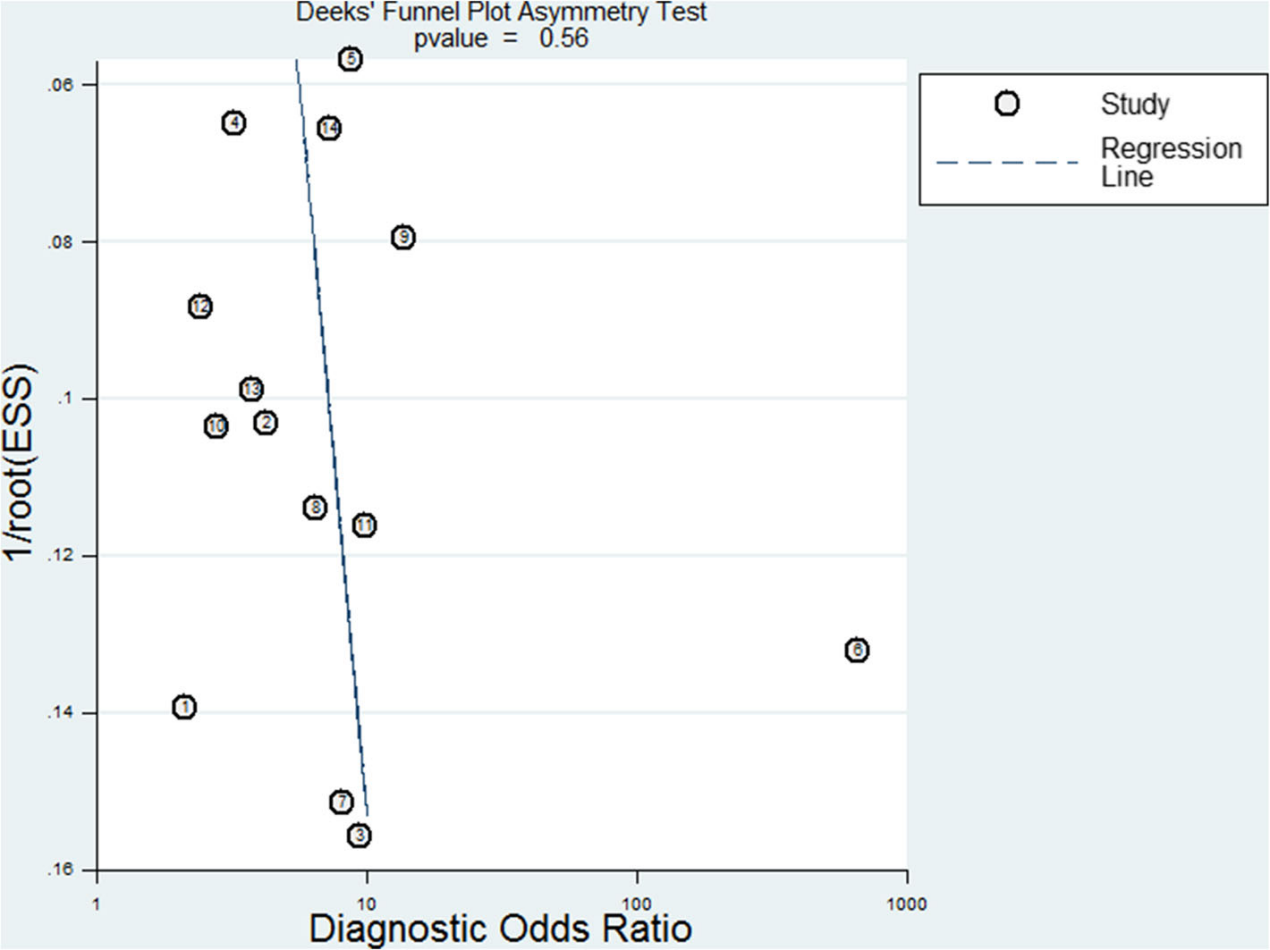
The Deek’s funnel plot for the assessment of potential publication bias

All diagnostic indices revealed high I^2^ values: sensitivity, 93.69; specificity, 91.55; PLR, 78.44; NLR, 91.50; and DOR, 99.85(*p* < 0.05 in all cases), indicating significant heterogeneity across all studies. Deeks’s funnel plot asymmetry test was used to evaluate the likelihood of publication bias among all 14 studies, and Deeks’s test identified low likelihood of publication bias, and with the *p* value of slope coefficient was 0.56 (Fig. 4).

## Discussion

To diagnose MPE accurately remains a clinical challenge, and the searching for useful biomarkers for MPE is still on the way. NSE is typical marker for cancers with neuroendocrine characteristic, especially for SCLC. Growing studies suggested that NSE is increased in MPE, and it may be a biomarker for MPE [32]. However, these studies gave different results. This study validated the diagnostic accuracy of NSE for MPE in 238 patients, which included the second largest patients that evaluated the diagnostic utility of NSE for MPE. In addition, we next performed a meta-analysis with 1896 subjects to make a full judgment of NSE for diagnosing MPE based on current available publications.

In this study, we enrolled 136 MPE patients, and we observed that both serum and pleural levels of NSE were higher in patients with MPE than in patients with BPE, even after adjustment by pleural protein. Pleural NSE shows a better diagnostic performance than serum NSE and pleural/serum NSE ratio, and its sensitivity and specificity were 59.56% and 83.33%, respectively. Pleural NSE showed a low sensitivity and a high rate of missed diagnoses, which may be due to only a limited proportion of NSCLC patients with neuroendocrine characteristic [7, 8]. Thus, the clinical value of NSE alone in screening MPE is limited. It may be more appropriate to use the combination of NSE and other tumor markers for diagnosing MPE [22–26].

The diagnostic performance of a serial of tumor markers for MPE, such as carcino-embryonic antigen, carbohydrate antigen 19–9, carbohydrate antigen 15–3, has been summarized by two meta-analysis, and studies suggested that one tumor marker alone doesn’t have not enough sensitivity to diagnose MPE, the combination of two or more tumor markers may increase the sensitivity and play more role in MPE diagnosis and management [33, 34]. In clinical utility, the results of NSE test should be used in conjugation with other tumor markers tests, and clinical information of patients, such as previous medical history, radiological findings.

For MPE with multiple causes (Lung adenocarcinoma, lung squamous cell carcinoma, SCLC, other etiologies), we noticed that both serum and pleural levels of NSE were highest in patients with SCLC, and NSE show a high diagnostic accuracy for SCLC-related MPE. Both serum and pleural NSE reach a sensitivity of 100% for SCLC-related MPE. Such results were also supported by Miédougé’s report [23]. These findings suggest the diagnostic performance of NSE may be tumor-subtype specific. Based on above findings, the NSE may not be used for screening MPE at the first choice. But for patients who were highly suspected for SCLC or neuroendocrine tumors, the examination of NSE may provide more valuable information.

To make a systemic assessment of the diagnostic performance of NSE for MPE, we performed an updated meta-analysis. A recent published meta-analysis has discussed the diagnostic role of NSE for MPE [35], however, it included only seven studies, and missed several studies. Thus, we made a more systemic literature search and updated this meta-analysis. In our meta-analysis, there were 1896 cases of patients, and the pooled sensitivity and specificity of NSE were 0.53 and 0.85, respective, confirmed our findings that NSE plays a role in confirming the diagnosis of MPE, rather than to screen MPE. The AUC was only 0.78, suggesting the diagnostic role of NSE for MPE is limited. Likelihood ratios are another indices of diagnostic accuracy, and PLR >10 or NLR <0.1 suggested high accuracy. In our meta-analysis, the PLR was 3.54, suggesting patients with MPE have about 3.5-fold higher possibility being pleural NSE measurement-positive. However, the NLR was as high as 0.56, which means that if the pleural NSE assay was negative, the chance that this patient has MPE was still as high as 56%, suggesting lack of differential ability. Anyway, the results of meta-analysis indicate that pleural NSE examination alone plays a limited role in diagnosing MPE.

Our study had several limitations. First, we only recruited 238 patients, and our meta-analysis only included 1896 patients, such limited number of patients may be not adequate for building final conclusions on the ability of NSE in diagnosing MPE. Second, only articles published in English were included, and there may be language bias exist, we may also miss the studies that not in the searched databases, both may bias the results. Further studies should include more patients from different centers to confirm the diagnostic role of NSE for MPE. The current NSE assay is with low sensitivity, it may be helpful to develop a novel method to examine NSE and increase the diagnostic accuracy. In addition, we found substantial heterogeneity among included studies. However, we didn’t investigate potential covariates with meta-regression analysis due to limited included studies.

## Conclusions

Taken together, the role of pleural NSE examination in diagnosing MPE is limited with low sensitivity. Our study suggests that the interpretation of NSE results should be in combination with the results of other tumor markers, and clinical data of patients. Further studies are needed to confirm our findings.

## Additional file

Additional file 1: Fig. S1. Serum and pleural levels of neuron specific enolase in patients standardized by pleural protein levels. After standardized by pleural protein levels, both serum and pleural levels of neuron specific enolase were higher than that in patient with benign pleural effusion. NSE: Neuron specific enolase; BPE: Benign pleural effusion; MPE: Malignant pleural effusion; LAC-MPE: Lung adenocarcinoma-malignant pleural effusion; LSCC-MPE: Lung squamous cell carcinoma- malignant pleural effusion; SCLC-MPE: Small cell lung cancer- malignant pleural effusion (TIFF 2523 kb)

## Abbreviations

ANOVA: Analysis of variance
AUC: Areas under the curve
BPE: Benign pleural effusion
DOR: Diagnostic odds ratios
FN: False negative
FP: False positive
MPE: Malignant pleural effusion
NLR: Negative likelihood ratios
NSCLC: Non-small-cell lung cancer
NSE: Neuron-specific enolase
PLR: Positive likelihood ratios
QUADAS: Quality Assessment of Diagnostic Accuracy Studies
ROC: Receiver operating characteristic
SCLC: Small-cell lung cancer
SROC: Summary receiver operating characteristic
TN: True negative
TP: True positive
