# Supplementary figures and images for: Is neuron-specific enolase useful for diagnosing malignant pleural effusions? evidence from a validation study and meta-analysis
Source: BMC Cancer. 2017 Aug 30;17:590. doi: 10.1186/s12885-017-3572-2 (PMC5575856; doi:10.1186/s12885-017-3572-2)

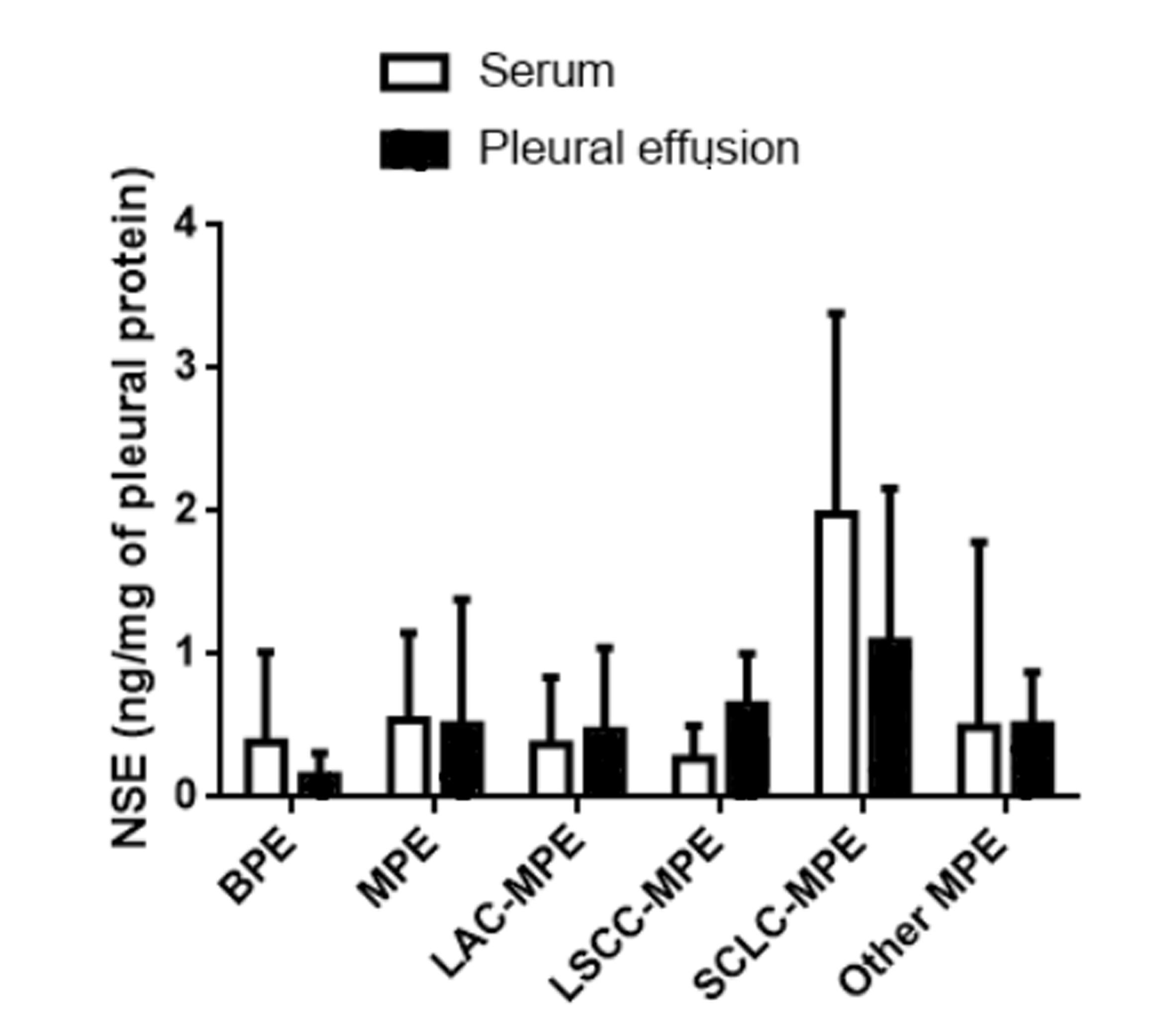

Supplement: Additional file 1: Fig. S1. — Serum and pleural levels of neuron specific enolase in patients standardized by pleural protein levels. After standardized by pleural protein levels, both serum and pleural levels of neuron specific enolase were higher than that in patient with benign pleural effusion. NSE: Neuron specific enolase; BPE: Benign pleural effusion; MPE: Malignant pleural effusion; LAC-MPE: Lung adenocarcinoma-malignant pleural effusion; LSCC-MPE: Lung squamous cell carcinoma- malignant pleural effusion; SCLC-MPE: Small cell lung cancer- malignant pleural effusion (TIFF 2523 kb) [file 12885_2017_3572_MOESM1_ESM.tif]
